# Health literacy of hospital patients using a linguistically validated Croatian version of the Newest Vital Sign screening test (NVS-HR)

**DOI:** 10.1371/journal.pone.0193079

**Published:** 2018-02-15

**Authors:** Sanja Brangan, Martina Ivanišić, Goranka Rafaj, Gill Rowlands

**Affiliations:** 1 Department of Educational Technology, Andrija Štampar School of Public Health, School of Medicine, University of Zagreb, Zagreb, Croatia; 2 Department of Anesthesiology, Reanimatology, and Intensive Care, University Hospital Center Osijek, Osijek, Croatia; 3 Study in Nursing, Technical College Bjelovar, Bjelovar, Croatia; 4 Institute of Health and Society, Newcastle University, Newcastle upon Tyne, United Kingdom; Università della Svizzera italiana, SWITZERLAND

## Abstract

The Newest Vital Sign (NVS) is a simple, quick and accurate screening test for health literacy (HL). It has been validated for different languages but, to date, not for the Croatian language. The aim of this study was to develop a linguistically validated Croatian version of the NVS and to use it at a later stage in a pilot study of health literacy assessment of hospital patients in Croatia. A full linguistic validation procedure was applied, including forward and backward translation, expert panel review, cognitive interview with 10 respondents from general population, and full involvement in the procedure of one of the screening test developers, the lead author of the NVS-UK version. HL testing on 100 hospital patients (55% women, median age 63.5 years) revealed 58% of patients had less than adequate HL level (scores less than 4), and mean NVS total score was 3.34. A positive significant association was observed between HL and educational level (p = 0.002). A high percentage of patients (92%) did not object to being tested for HL by their primary care physician or in hospital, and 99% of patients would recommend HL testing among patients in general. The respondents' positive views on HL testing and mean completion time of 4 minutes indicate that the Croatian version of the NVS (NVS-HR) could be recommended for use in both clinical and research settings in Croatia.

## Introduction

Various tests of health literacy (HL) have been developed over the past years as a result of the finding that poor HL may be a stronger predictor of a person's health than age, income, employment status, education level, and race [1]. Additionally, HL has been shown to impact patients' knowledge, health behaviours, health outcomes, and medical costs [2]. A systematic review on low health literacy and health outcomes from 2011 [3] showed that low health literacy is consistently associated with more hospitalizations; greater use of emergency care; lower receipt of mammography screening and influenza vaccine; poorer ability to demonstrate taking medications appropriately; poorer ability to interpret labels and health messages; and, among elderly persons, poorer overall health status and higher mortality rates. Data on the US population [2] show that nearly half of the adult population has difficulty understanding and using health information. In Europe, a study on HL in eight EU countries [4] showed that people with low HL have a higher prevalence of long-term conditions, which in turn are more likely to be limiting, and that low HL level is significantly associated with worse self-assessed health. Research on HL in Croatia is still in its infancy, with no studies published at the national level [5] and previous published studies either using a nonvalidated instrument [6] or focusing mostly on readability, comprehension or informed consent issues [5,7–11].

There are multiple definitions of health literacy. One of the most commonly used is 'the personal, cognitive and social skills which determine the ability of individuals to gain access to, understand and use information to promote and maintain good health' [12]. This definition includes three 'levels' of HL: basic or functional; communicative or interactive; and critical HL [12]. Although the levels are not presented as hierarchial, it is reasonable to assume that basic functional HL and numeracy skills are important in forming the basis for more complex competencies [13].

The Newest Vital Sign (NVS) is a test of functional health literacy, assessing reading comprehension and numeracy skills. It was first developed for US population in 2005 [13], and the UK version (NVS-UK) was developed in 2013 [14], with adaptations for the European context. The NVS is a 6-item test based on the ability to read and apply information from an ice cream nutrition label; it takes three minutes to administer; and the scoring enables identification of three groups of patients: likely marginal/inadequate HL, likely limited HL, and likely adequate HL [13,15,16]. The NVS (US version) scores correlate with the scores of the widely used but lengthier validated instrument for HL assessment, the Test of Functional Health Literacy in Adults–TOFHLA [17], which served as the reference standard. In clinical settings it is recommended that the NVS scores are recorded in the patient's chart, so that future communication can be tailored to ensure patient understanding [16].

Ever since the original NVS was developed by Weiss et al. [13] for the US population, both in English and Spanish, there have been translations/adaptations of the NVS for other languages. Review of the literature published in English on details of translation procedures lists the following language versions of the NVS: Turkish [18], Dutch [19,20], Portuguese [21], Arabic [22,23], Japanese [24], Italian [25], Malay [26], and Brazilian Portuguese [27]. The report on HL survey in eight EU countries [4] does not describe translation procuredure of the NVS for Bulgarian, German, Greek, and Polish versions although data on HL results are included for those countries as well; however, a German language version is briefly described by Berens et al. [28]. A Hungarian NVS version is described in a paper in Hungarian language [29].

The usually adopted but complex procedure of translation/cultural adaptation–termed also *linguistic validation*, of patient-reported outcome (PRO) instruments is described by Acquadro et al. [30]. This includes initial engagement with the instrument developer, forward and backward translation, review of the reconciled version with the developer of the original instrument, cognitive interviews, and expert panel review. To the best of our knowledge, based on literature review, there is no test of functional health literacy developed in or translated into Croatian language that has undergone full linguistic validation procedure. The aim of this study was to develop a linguistically validated Croatian version of the NVS health literacy test and to use it at a later stage in a pilot study of health literacy assessment of hospital patients in Croatia.

## Materials and methods

The Croatian version of the NVS instrument (NVS-HR) is based on the NVS-UK version, which adapted the NVS-US for the European context. The linguistic validation process followed the procedure as described by Acquadro et al. [30] for PRO instruments. The NVS-UK version was translated into Croatian by two independent professional translators, native speakers of Croatian, who were given an instruction to take into account the Croatian style of ice cream nutrition labels found in the market, which are in line with the EU and Croatian regulations [31], but at the same time to keep the Croatian translation as close as possible to the source text of the NVS-UK, and to check the NVS-US for further assistance, if needed. One forward translator was also provided with the Italian translation of the NVS-UK and the Spanish translation of the NVS-US for further assistance, since they were also fluent in these two languages. A panel of five experts covering the fields of linguistics, health literacy, public health, medicine (neurology), nutrition, and nursing, reviewed the two forward translations taking into account the same instructions as given for the forward translation step and prepared a reconciled version by agreeing on the best translation option for the entire NVS test. The reconciled version was backtranslated by a professional translator, native speaker of English and fluent in Croatian. The backtranslated version was reviewed by a panel of 3 out of 5 experts, as well as by the developer of the NVS-UK. Cognitive interviews were done face-to-face by a trained interviewer with 10 respondents recruited from general population in an urban area by a medical nurse, with the inclusion criteria of both genders, varying age and occupation, lower educational level (primary and secondary school, that is, up to 12 years of schooling). Respondents were given a hard copy of the NVS ice cream nutrition label to read to themselves, then answered the NVS questions read out loud by the interviewer, and afterwards discussed the instrument with the interviewer focusing on: general impression (length and difficulty of the instrument); questions about which parts/words/phrases they did not understand; what each NVS item meant to them in their own words, with examples; how they reached the answers to NVS questions; and suggestions for modifications. The cognitive interview results were again reviewed by 3 out of 5 experts and the developer of the NVS-UK. The final version (NVS-HR) was reached when there were no more suggestions for modifications in the linguistic validation step.

The NVS-HR was then used to assess HL within a pilot study involving a convenience sample of 100 hospital patients in an urban area of eastern Croatia, a part of the country that was heavily affected by war in the 1990s, with a high unemployment rate (12.1%, July 2016) [32], and increasing emigration of young people. This part of the study was performed in the largest hospital of Osijek-Baranja County, the University Hospital Center Osijek, with the reported total bed capacity of 986 for stationary patients in 2016 [33]. Exclusion criteria were: patients under 18 years of age and patients from departments that provide care to mental patients since no mental capacity assessments were planned. The hospital departments included in the study, with the respective bed capacity as reported by the hospital nurse, were: Surgery (185), Internal Medicine (150), ENT (26), Orthopedics (40). Taking into account exclusion criteria and expecting occasional objective barriers to NVS testing, such as postoperative pain, high percentage of patients without glasses [10], the approachable realistic number in each department was estimated as 50, 50, 20 and 10 patients, respectively. Expecting a relatively high response rate, we set the target number of 100 patients in the sample for this pilot study.

The study was approved by the hospital ethics committee, Committee for Ethical and Profession Issues of Medical Nurses and Technicians, University Hospital Center Osijek, and each patient signed an informed consent form. All patients from the four hospital departments were approached consecutively until the target number of 100 was reached. The testing was done in January 2017, by a trained interviewer, a medical nurse employed in the hospital. Data were collected on patient sociodemographic characteristics and their overall opinions on HL testing, with questions constructed for the purpose of the study. Each patient was given a laminated copy of the NVS-HR ice cream nutrition label, and the NVS-HR questions were read out loud to the patient. No use of a calculator or any supporting materials was allowed. The NVS-HR testing completion time was recorded for each patient.

Reflecting the scoring in current versions of the NVS, the NVS-HR total scores (0–6) were interpreted as follows: ≥4 = adequate HL; 2–3 = intermediate HL; 0–1 = low HL. Question 7, since considered a subquestion of Question 6 also in the NVS-UK, when answered correctly, was interpreted as 'Question 6 answered correctly'.

All statistical analyses were performed using the Statistical Package for the Social Sciences (SPSS version 22.0; Chicago, IL, USA) software. Collected data are described by frequencies and percentages. We used means, standard deviation (SD), and median values to summarize the respondents' characteristics and performance on the test. For categorical variables, Fisher's exact test was used to compare the groups. Values of p < 0.05 were considered statistically significant.

## Results

### Stage 1 –Linguistic validation of the NVS

**Fig 1:**
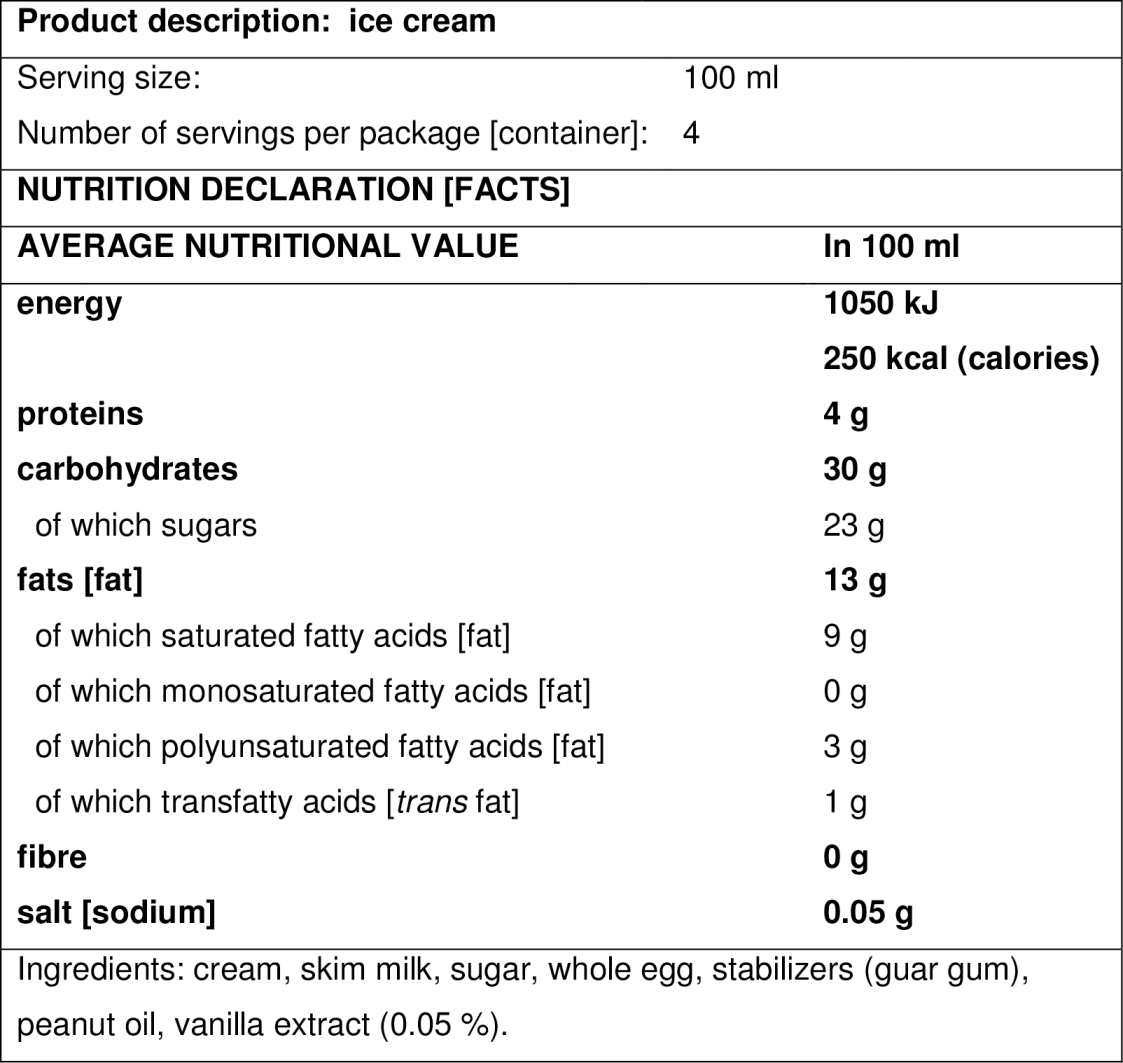
NVS nutrition label backtranslated from Croatian.

The backtranslation of the nutrition label as worded in the Croatian reconciled version is given in Fig 1. The backtranslator's second option for some words is presented in square brackets.

Analysis of consideration points, made by the expert panel upon backtranslation of the reconciled version, resulted in keeping the NVS text in Croatian unmodified since reflecting well the document in English and complying with the standards used in Croatian nutrition labels. The only problem arose with Question 2 (Q2) wording “how many ice creams maximum are you allowed to eat?”, which could be interpreted as ‘how many containers/packages’, so it was changed to: “what maximum amount of ice cream may you eat?”.

Cognitive interviews with 10 respondents from general population revealed that the respondents considered the NVS test short enough and not complicated, although requiring some concentration and mathematical skills. Some respondents asked for some questions to be repeated, which was done, and one asked for a calculator, which was not allowed. Demographic characteristics and NVS scores for 10 respondents in the cognitive interview step are given in Table 1.

**Table 1:** Demographic characteristics, completion time, and NVS scores of 10 respondents in cognitive interview step.

No.	Age	Gender	Educational level (total years of schooling)	Occupation	Residence	Completion time (min.)	NVS score
1	46	F	Secondary school (11)	unemployed shoe maker	urban	4.31	6
2	38	F	Primary school (8)	cleaner	urban	9.05	3
3	32	F	Secondary school (11)	hair dresser	rural	2.25	6
4	18	F	Secondary school (12)	unemployed trade worker	rural	6.37	5
5	21	F	Secondary school (12)	secretary	urban	8.28	5
6	55	M	Secondary school (11)	self-employed in farming business	rural	8.45	6
7	50	M	Secondary school (12)	retired army officer	rural	3.48	5
8	43	M	Secondary school (12)	construction worker	urban	4.15	5
9	24	M	Secondary school (12)	shop assistant	urban	2.45	6
10	68	M	Primary school (4)	retired worker in agriculture	rural	7.32	5

The NVS nutrition label was well understood, although some respondents found the fat subtypes confusing, suggested one word only for calories, or did not understand whether ‘guar gum’ was related to rubber gloves and therefore to allergic reaction mentioned in the test. The NVS test questions raised a few concerns. Some respondents miscalculated Question 1 either by confusing ‘portion’ with ‘package’ or not knowing where to find the correct values, and one respondent mentioned not necessarily reacting to peanuts. The interviewer also noted that for some respondents it was difficult to calculate when hearing a question just once so they miscalculated, or they focused on numbers only or some words only forgetting e.g. a difference between serving size and package. The expert panel and the developer agreed that modifications suggested by respondents were not needed since they were either irrelevant or would actually change the discriminatory power of the test, except for one wording: ‘guar gum’ (Croatian: ‘guar *guma*’) may be closely related to ‘rubber gloves’ (Croatian: ‘*gumene* rukavice’–made of gum), which made some respondents think ‘if allergic to rubber gloves, it’s not safe to eat this ice cream’. Therefore, this confusing wording of ‘guar gum’ was modified to ‘guar mixture’.

### Stage 2 –Health literacy testing among hospital patients

A total of 144 consecutive patients from the four hospital departments were considered for HL testing using the NVS-HR, to reach the target sample size of 100 participants. For the 44 patients not included in the sample, since no informed consent could have been obtained, the following reasons were given: not having reading glasses (15); problems with eyesight (2); not having a hearing aid (1); severe medical condition–poor general health (8), mental illness (3), postoperative pain (7); illiterate patients (2); refusal to participate–because of no personal gain from the study (1), no clear purpose of study results (2), not stating a reason (3). The final sample included 49 patients from Internal Medicine department, 34 from Surgery, 10 from ENT, and 7 from Orthopedics.

Of the total 100 patients who participated in the study, 55% were women, 44% over 65 years of age (median age 63.5 years, range 18–84), 59% retired, 58% with secondary level education (11 or 12 years of schooling), 67% with very low or no income, 53% with self-reported chronic condition, and 69% with BMI ≥ 25 (32% overweight, 37% obese). Patient sociodemographic and baseline characteristics are given in Table 2.

**Table 2:** Patient sociodemographic and baseline characteristics (n = 100).

Variable		%
Gender	Male	45
	Female	55
Residence	Urban	64
	Rural	36
Age (years)	< 45	15
	45–65	41
	> 65	44
Education	Without or incomplete primary school	8
	Primary school	22
	Secondary school	58
	College or more	12
Employment status	Student / training	3
	Unemployed	5
	Housewife	8
	Self-employed	2
	Retired	59
	Employed (full time)	23
Monthly income* [34]	No income	3
	< 3000 HRK	64
	3000–6000 HRK	24
	6000–12000 HRK	9
BMI	Underweight (<18.5)	3
	Normal weight (18.5–24.99)	28
	Overweight (25–29.99)	32
	Obesity (≥30)	37
Self-reported chronic condition	No chronic condition	47
	Diabetes	21
	Gastritis	5
	Other	27

HRK = Croatian kuna (EUR 1 = HRK 7.6; January 2017)

* Average net monthly income in Croatia for January 2017 = HRK 5,895

**Fig 2:**
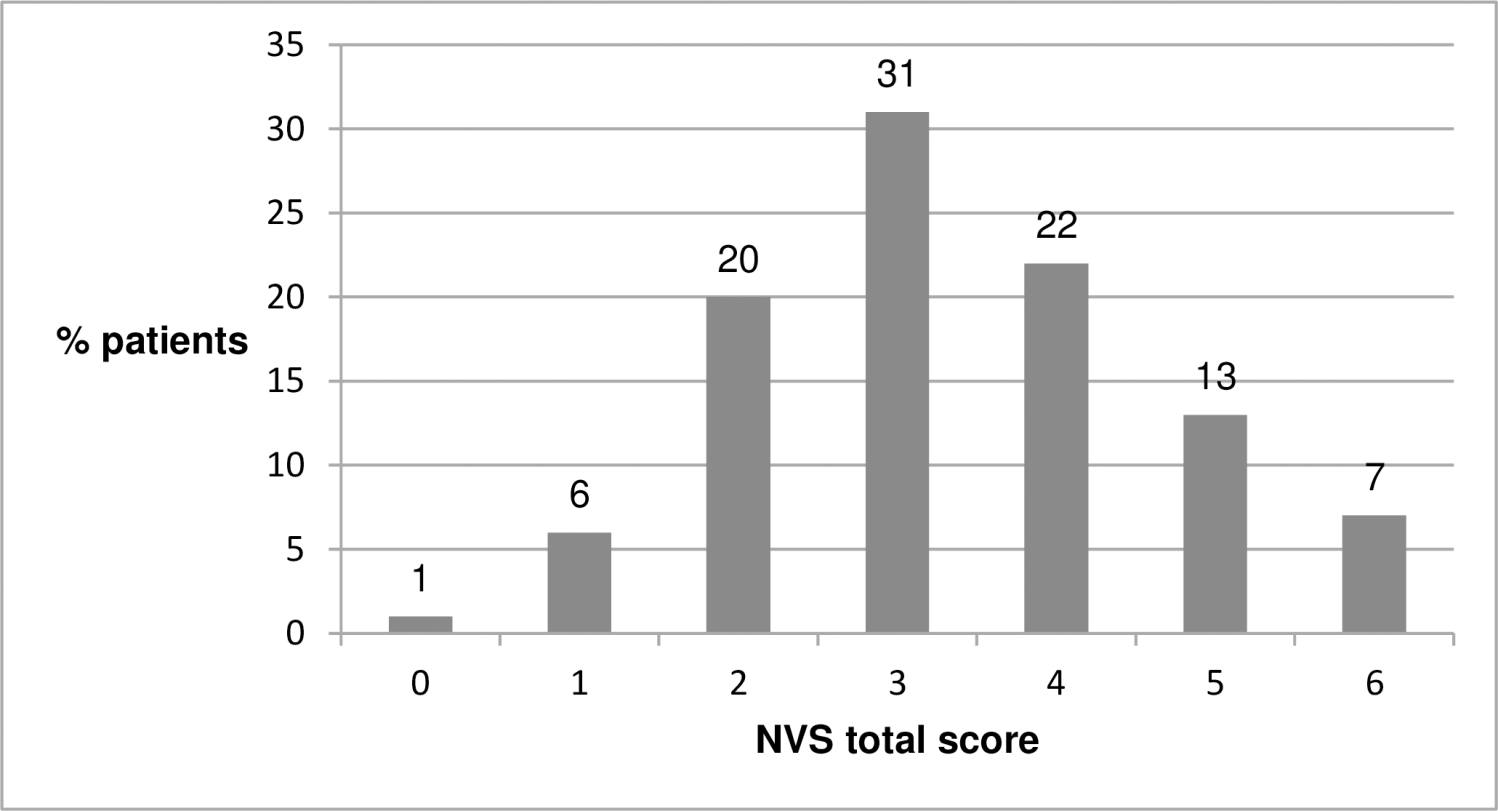
NVS total score with percentage of patients.

Mean completion time was 4.2 minutes (SD = 1.2 minutes), with range 1.9–8.8 minutes. Mean NVS total score was 3.34 (SD = 1.3), with most patients (31%) scoring 3 points. The NVS total scores with percentage of patients per score are shown in Fig 2.

Percentage of patients who gave correct answer to each of the 6 NVS questions is given in Table 3, with backtranslation of each NVS-HR question. Q7, as a subquestion of Q6, when answered correctly, was interpreted as 'Q6 answered correctly'. The easiest question was Q5 and the most difficult was Q1 (only 24% correct), where most patients, 59 out of 76 who answered incorrectly, replied incorrectly '250 kcal'.

**Table 3:** Percentage of correct answers to each NVS-HR question.

NVS question	% of correct answers
Q1. How many calories (kcal) will you consume if you eat the entire package?	24
Q2. If you are advised not to eat more than 60 grams of carbohydrates for dessert, what maximum amount of ice cream may you eat?	33
Q3. Imagine your doctor advising you to reduce the amount of saturated fat in your diet. You normally consume 42 g of saturated fat daily, which includes one serving of ice cream. If you stop consuming ice cream, how many grams of saturated fat will you be consuming every day?	47
Q4. If you normally consume 2500 calories every day through food, what percentage of your daily intake of calories (kcal) will you consume if you eat one serving of ice cream?	74
Q5. Imagine that you are allergic to the following substances: penicillin, peanuts, rubber gloves and bee stings. Is it safe for you to eat this ice cream?	80
Q6. [If the answer to Question 5 is "no"]: Why not?	76
(Q7. [If the answer to Question 6 is "Because I could have an allergic reaction", ask:] Why would you have an allergic reaction?)	

Adequate HL level was found in 42% of patients; 51% had intermediate HL level, and 7% had low HL level. HL levels by sociodemographic and baseline characteristics are shown in Table 4. A positive significant association was observed between HL level and educational level, showing that patients with higher education scored significantly higher on the NVS test. Namely, no patient with high educational level had low HL, and 91.7% of those patients had adequate HL. Statistically significant differences were observed in employment status as well but the number of patients in many analyzed groups was too low to draw any firm and clear conclusions. Statistically significant associations between HL levels and other sociodemographic or baseline characteristics were not found.

**Table 4:** Health literacy levels by sociodemographic and baseline characteristics (n = 100).

Characteristic	Health literacy level (%; n)			P-value
	Low	Intermediate	Adequate	
**Age group (years)**				P = 0.130
< 45	0.0% (0)	33.3% (5)	66.7% (10)	
45–65	12.2% (5)	46.3% (19)	41.5% (17)	
> 65	4.5% (2)	61.4% (27)	34.1% (15)	
**Gender**				P = 0.269
Male	11.1% (5)	53.3% (24)	35.6% (16)	
Female	3.6% (2)	49.1% (27)	47.3% (26)	
**Educational level**				P = 0.002
Low (no education; incomplete or complete primary school)	10.0% (3)	66.7% (20)	23.3% (7)	
Medium (secondary school)	6.9% (4)	51.7% (30)	41.4% (24)	
High (college or above)	0.0% (0)	8.3% (1)	91.7% (11)	
**Residence**				P = 0.167
Urban	3.1% (2)	53.1% (34)	43.8% (28)	
Rural	13.9% (5)	47.2% (17)	38.9% (14)	
**Employment status**				P = 0.036
Student / training	0.0% (0)	0.0% (0)	100.0% (3)	
Unemployed	20.0% (1)	20.0% (1)	60.0% (3)	
Housewife	0.0% (0)	62.5% (5)	37.5% (3)	
Self-employed	50.0% (1)	0.0% (0)	50.0% (1)	
Retired	8.5% (5)	59.3% (35)	32.2% (19)	
Employed (full time)	0.0% (0)	43.5% (10)	56.5% (13)	
**Monthly income**				P = 0.390
No income	0.0% (0)	100.0% (3)	0.0% (0)	
< 3000 HRK	7.8% (5)	53.1% (34)	39.1% (25)	
3000–6000 HRK	4.2% (1)	37.5% (9)	58.3% (14)	
6000–12000 HRK	11.1% (1)	55.6% (5)	33.3% (3)	
**BMI**				P = 0.583
Underweight (<18.5)	33.3% (1)	33.3% (1)	33.3% (1)	
Normal weight (18.5–24.99)	3.6% (1)	57.1% (16)	39.3% (11)	
Overweight (25–29.99)	9.4% (3)	53.1% (17)	37.5% (12)	
Obesity (≥30)	5.4% (2)	45.9% (17)	48.6% (18)	
**Self-reported chronic condition**				P = 0.901
No chronic condition	6.4% (3)	46.8% (22)	46.8% (22)	
Diabetes	9.5% (2)	52.4% (11)	38.1% (8)	
Gastritis	0.0% (0)	80.0% (4)	20.0% (1)	
Other	7.4% (2)	51.9% (14)	40.7% (11)	

Patients also replied to several questions on HL testing in general. A high percentage of patients (92%) did not object to being tested for HL by their primary care physician or in hospital; three patients said they would feel shame, two patients said it would be a waste of the physician's time, and three gave no reason for their objection. Also, 99% of patients would recommend HL testing among patients in general, and 98% were of the opinion HL testing results would help physicians and medical nurses in their communication with patients. When asked about reading ice cream or other food labels, 89% mentioned never reading ice cream nutrition labels, and 62% mentioned never or rarely reading food labels.

## Discussion

The development of robust HL tests is lengthy and complex, as reflected in the description of the development of the original NVS (US version) [13]. The cultural and linguistic adaptations for another country and/or language need to undergo a rigorous procedure for the test to be acceptable and useful [19,20]. This implies the process of cultural adaptation as well as translation where needed. The Croatian version of the NVS underwent full linguistic validation procedure as described by Acquadro et al. [30] for translation/cultural adaptation of PRO instruments, before it was used for HL testing of 100 hospital patients. Since the rigorous procedure included forward/backward translations by professional translators experienced in the procedure, along with pre-testing on general population, expert reviews, and full involvement of the lead author of the NVS-UK version, only minor modifications were required to reach the final text version. The procedures adopted for the NVS versions in other countries have varied from a Delphi technique [14,20], full [19,21,23,27] or partial linguistic validation [22,24,25], to forward and backward translation only [18,26]. When the step of cognitive interviews is excluded, valuable information may be missing on why some NVS questions result in incorrect answers. Cognitive interviews in this study revealed why some respondents miscalculated in Question 1 (Q1)–confusing 'portion' with 'package', which was done by 59 hospital patients tested for HL as they replied incorrectly '250 kcal'. Q1 was identified as the most difficult; the same, along with Q3, was found in a study among Canadian immigrants [35], and Q3 alone, considered very long and complex [20] was the most difficult in some other language versions [18,36] that at the same time found Q5 the easiest, identical to this Croatian study.

Cultural issues with the NVS test related to unfamiliarity with nutrition labels in the respondents' daily activities have been described [19,26] and also observed in this study with hospital patients. When asked about reading ice cream or other food labels, 89% of patients mentioned never reading ice cream nutrition labels, and 62% mentioned never or rarely reading food labels. However, we feel that a test based on understanding and use of a nutrition label remains a valid screening tool for HL as it directly relates to an activity required for healthy eating, and tests the abilities to navigate through text, find the relevant information, and draw conclusions leading to potential health-related decision-making. Also, the NVS allows clinicians and health administrators to rapidly assess HL in their patients–within 3 minutes [13], in 3–6 minutes [21], or around 4 minutes [22] as found in this study. Some language versions did require longer mean completion times– 6.28 [18] and 10.8 [23], but HL testing in Iraq was self-administered, allowing the participants to read the entire text on their own, which perhaps yielded such long completion times. Although a study on the use of the NVS has indicated to a limited practicality of this test in the elderly population with the mean completion time of 11 minutes [37], 25% of the participants over the age of 65 in this Croatian study completed the test within 4 minutes, and only three required more than 6 minutes.

Although different methodologies have been used for development of the different language versions, and the sample size and profiles have varied in the NVS validation studies, results of HL testing may be compared internationally, but to a certain degree and with caution. HL testing with NVS-HR on 100 hospital patients identified 42% of patients with adequate HL, 51% with intermediate HL, and 7% with low HL (mean total score 3.34). A very similar mean score of 3.4 was observed with both NVS-US English version [13] and the NVS-UK [14], and slightly lower (3.2) in Kuwait [22] and Iraq (3.02) [23]. On the other hand, very low mean scores were observed in Spanish [13], Dutch [19], Turkish [18], and Japanese [24] study groups– 1.6, 1.8, 2.6, and 2.1, respectively. Also, the NVS-HR mean score was much lower than with NVS-D [20] and NVS-PT [21] of 4.2 and 4.4, respectively. However, the NVS-D testing [20] was partly done online on respondents who had access to and were familiar with the use of the Internet, and were allowed to read the NVS questions and use a calculator, whereas the NVS-PT [21] testing was done mostly on students. Additionally, the NVS-HR mean score is similar to the average EU score of 3.5, as reported in a study on HL among 8 EU countries [4], where adequate HL level was found in 55.3% of the total study population. Interestingly, with the NVS-BR [27], adequate HL was found in 48.7% of adult pharmacy clients compared to 33.5% of public school teachers. The lower HL levels among teachers compared to the general population are explained by the fact that the teachers may have not paid enough attention to the NVS since they were given another lengthy instrument to complete first, leading the authors to note that the NVS scores might be impacted by the number of items in multiple-scale surveys.

HL testing with NVS-HR showed a significant positive association between educational level and HL, reflecting finding from a systematic review [38], the NVS-UK [14], a HL survey of 8 EU countries for the general sample [4], the NVS-D [19,20], and the Turkish study [18]. Whilst the associations between HL and education level appear to be consistent across countries, it is important to note the international variations in associations between other socio-economic and demographic determinants of health and HL. For instance, no statistical association between HL and gender was found with the NVS-HR, and neither in a systematic review of US studies [38] or some NVS language versions where it was measured and reported [4,13,23,27]. On the other hand, the Turkish study [18] found the lowest NVS scores in female participants whereas the NVS-D scores [20] were significantly higher in the female study population. As to age, both of those studies found older participants to have lower NVS scores, the same as the Portuguese [21] and the Brazilian Portuguese study [27], but not with the NVS-HR. It is likely that these differences reflect differences in national education and health systems, and national culture and context [4,39], meaning that an understanding of HL within different countries requires local measurement and exploration of the associations with socio-economic and demographic factors, and cannot be imputed from other national settings [39].

Weiss et al. [13] cautioned that patients with probable marginal or inadequate literacy (NVS scores below 2) cannot be reliably identified by clinicians if asked only about their educational level, as education does not always predict literacy. Although HL testing with NVS-HR showed significant positive association between educational level and HL level, educational level alone would not identify each patient with limited literacy–for example, 6.9% of those with medium educational level had low HL, and 23.3% with low educational level had adequate HL level, so an objective HL test applied in clinical practice, especially when it takes around 4 minutes, should be considered a better and more precise predictor. Weiss et al. consider the NVS test as 'a quick and accurate screening test for limited literacy' suitable for primary health care settings [13]. Authors of the NVS-UK version consider this instrument a simple and accurate predictor of health literacy skills, which can be administered by both clinical and non-clinical personnel, and it has a potentially valuable role in improving clinical practice and patient communication [14]. Furthermore, some studies have shown that healthcare providers, including physicians and nurses, overestimate their patients' health literacy levels, but also that they have limited knowledge and skill related to health literacy assessment. For example, one NVS testing showed that 63% of patients had a high likelihood of limited HL and 22% of patients had a high likelihood of adequate HL, whereas nurses reported 19% and 68%, respectively [40]. The potential use of a quick, sensitive and specific measure of HL in clinical practice has been recognized; however, there has been anxiety about the routine use in clinical practice due to concerns about the impact of stigma on the patients [41], and hence on the doctor-patient relationship. This has resulted in suggestions that a better approach is one of 'universal precautions', where the focus is on developing a health service accessible to all, regardless of HL capacities [42]. Whilst this is a commendable approach, it is likely that financial and time constraints may hamper or preclude such an approach. Our finding that patients in this Croatian study had no objections (92%) to HL testing, with as many as 99% recommending HL testing among patients, and 98% being of the opinion that HL testing results would help physicians and medical nurses in their communication with patients, is supported by Ryan et al. [43]. This brings an additional option to health care providers in resource-constrained settings such as Croatia.

Among the limitations of this study, we would include a relatively small sample size (n = 100), which in turn yielded a relatively high percentage of the participants in retirement (59%), not formally employed (75%), with low income (67%), being overweight or obese (69%), and even of the age ≥45 years (85%). However, this was a convenience sample where the patients were approached consecutively in the four hospital departments, so it reflected the actual situation both in the hospital and the area. Also, this pilot study was limited to a single hospital center but it is the only one in this eastern county and among the five largest hospitals in Croatia. Selection of the hospital setting for participant recruitment may be justified by the fact that a large hospital center would admit patients from different urban and rural smaller primary care units in the county. On the other hand, any generalizations of the findings for the general population should be made carefully considering the selection of participants and the small sample size. Finally, data on ethnicity were not collected, although ethnicity plays an important role in HL testing worldwide [3,4], due to the fact that 90% of the population both in Croatia and the city of Osijek declare themselves as Croats [44].

## Conclusion

This study developed the Croatian version of the NVS health literacy screening tool, based on the UK version since adapted for the European context, and following a full linguistic validation procedure. HL testing with NVS-HR on 100 hospital patients identified 42% of patients with adequate HL, found the mean total NVS score of 3.34, and statistically positive association of HL with educational level. These findings are in line with the results of other studies describing the development of the NVS for other languages. Results also showed a short average completion time of the NVS-HR of 4 minutes, and the exceptionally high percentage (99%) of study participants who would recomment HL testing among patients in general. Considering the fact that the NVS is considered a quick and simple but also an accurate screening test for health literacy, we recommend the NVS-HR for HL testing in clinical settings in Croatia. As in other countries, the NVS-HR is also likely to be a useful tool for researchers investigating the impact of health literacy on health and illness.
